# The current state of xenotransplantation

**DOI:** 10.1007/s13353-014-0261-6

**Published:** 2014-12-07

**Authors:** J. Zeyland, D. Lipiński, R. Słomski

**Affiliations:** 1 Department of Biochemistry and Biotechnology, Poznan University of Life Sciences, Poznan, Dojazd 11, 60-632 Poland; 2 Institute of Human Genetics, Polish Academy of Sciences, Poznan, Strzeszynska 32, 60-479 Poland

**Keywords:** Pig, Primate, Rejection, Genetic engineering, Xenotransplantation

## Abstract

Pigs as a source of grafts for xenotransplantation can help to overcome the rapidly growing shortage of human donors. However, in the case of pig-to-human transplantation, the antibody-xenoantigen complexes lead to the complement activation and immediate hyperacute rejection. Methods eliminating hyperacute rejection (HAR) include α1,3-galactosyltransferase (GGTA1) inactivation, regulation of the complement system and modification of the oligosaccharide structure of surface proteins. The humoral immune response control and reduction of the risk of coagulation disorders are the priority tasks in attempts to overcome acute humoral xenograft rejection that may occur after the elimination of HAR. The primary targets for research are connected with the identification of obstacles and development of strategies to tackle them. Because of the magnitude of factors involved in the immune, genetic engineers face a serious problem of producing multitransgenic animals in the shortest possible time.

## Introduction

The shortage of human organs for transplantation has motivated scientists to consider how new technologies such as genetic, tissue and cellular engineering might be used to improve the function or replace failing organs. As statistics show, a demand for organ transplantation, measured by the number of patients on waiting lists, has increased in the last decade. In 2011, according to the Annual Report of Organ Procurement and Transplantation Network (OPTN) in the United States of America, there is one registered donor per every ten patients awaiting transplantation. The situation is very dynamic: the demand for kidney transplants has doubled in the last decade, whereas at the same time that for lung transplants has nearly halved. Improvements in the system of organ registration and distribution, promotion of healthy lifestyle to improve the physical fitness of the society, decreasing the frequency of chronic graft rejections thanks to reduced toxicity of immunosuppressive treatments and inducing tolerance are the most important challenges of modern transplantology. However, all these measures are insufficient to improve the short supply of potential organs and transplantation tissues. This dramatic situation has resulted in studies on alternative sources of organs.

The concept of cross-species transplantation is not a new idea. Clinical attempts in this field have been conducted during the past 300 years (Cooper 2012). The phylogenetic proximity of non-human primates and humans is the strongest argument for xenotransplantation, although at the same time it carries the greatest risk of xenozoonoses. An unlimited availability, good breeding potential, short period to reproductive maturity, relatively short pregnancy, a high number of offspring, anatomical and physiological similarities to humans, low risk of infection transfer with availability of specific pathogen-free animals indicate pigs as animal donors of tissues and organs for transplantation (Cooper et al. 2002). However, we must remember that the large phylogenetic distance will induce numerous perturbations after xenotransplantation.

## Immunobiology of xenotransplantation

Transplantation of wild type pig organs into non-human primates activates the complement cascade by binding naturally occurring preformed antibodies to carbohydrate Galα(1,3)Gal epitopes on the surface of porcine endothelial cells (ECs) causing hyperacute rejection (HAR) (Lexer et al. 1986). Type I (nontranscriptional) activation is rapid and leads to ECs retraction, expression of P-selectin (CD62P), secretion of the platelet activating factor (PAF) and loss of the antithrombotic phenotype (Cho et al. 2012). Immunoglobulin G (IgG) after specific binding to target carbohydrate antigens on endothelial cell membranes induces C3b production and complement-mediated vascular collapse. Galvao et al. showed that rabbit-to-swine multivisceral xenotransplants undergo moderate HAR in the liver, but the other organs suffer from severe HAR. An accumulation of IgG, especially in the vascular endothelium, was observed and caused edema, hemorrhage, thrombosis, myositis, fibrinoid degeneration and necrosis (2012). Free radicals, especially reactive oxygen species (ROS) and nitric oxide species (NOS), contribute to injury, rejection and dysfunction of the xenotransplanted organs. HAR observed in a xenogeneic perfused liver was triggered by the complement system only in the presence of leukocytes and free radical formation (Ngo et al. 2013).

Results of protocol biopsies demonstrated that the degree of subsequent acute humoral xenograft rejection (AHXR) was reduced in hDAF transgenic animals lacking antibodies against Galα(1,3)Gal epitopes compared with control animals. Intravenous administration of GAS914, a soluble, polymeric form of a Galα(1,3)Gal trisaccharide, significantly reduced xenoreactive antibodies, therefore was able to prevent not only hyperacute rejection, but also acute vascular rejection at its beginning (Zhong et al. 2003). Although pigs that lack Galα(1,3)Gal particles ensure prolonged survival of xenografts, other epitopes are still expressed and may induce AHXR as subsequent rejection.

Vascularized grafts are lost because of AHXR characterized by type II (transcriptional) endothelial cell activation, thrombosis with fibrin deposition and cellular infiltration. Natural killer cells (NK), monocyte/macrophages and neutrophils play a role in AHXR, but only the importance of the NK is clear (Inverardi et al. 1997; Fox et al. 2001; Rieben and Seebach 2005). Park et al. in their study showed that the activity of GGTA1 in the lungs, liver, spleen and testes excluding the brain and heart of heterozygous GTKO (α1,3-galactosyltransferase gene-knockout) pigs was significantly decreased when compared to the controls. The same pigs had more sialylα2,6- and sialylα2,3-linked glycan than the control. The heart, liver and kidneys of heterozygous GTKO pigs had much higher N-glycolylneuraminic acid (Neu5Gc) contents than the control. Based on these results the authors indicated Neu5Gc and named it the Hanganutziu-Deicher (H-D) antigen, not synthesized in humans, as crucial for overcoming the next acute immune rejection in pig-to-human xenotransplantation (2012a, b). The cytidine monophosphate-N-acetylneuraminic acid hydroxylase (CMAH) catalyzes the conversion of CMP-N-acetylneuraminic acid (CMP-Neu5Ac) into its hydroxylated derivative CMP-N-glycolylneuraminic acid (CMP-Neu5Gc). Lutz et al. showed that the humoral barrier to xenotransplantation was reduced to a greater degree in the case of pigs lacking both CMAH and GGTA1 gene activities than it was with pigs lacking only GGTA1 (2013). At the same time, Kwon et al. produced both mono- and bi-allelic CMAH knock-out (CMAH-KO) miniature pigs with the use of zinc finger nucleases, which showed detectable decreased transcript levels of the H-D antigen. CMAH-KO pigs were healthy and showed no signs of abnormality or off-target mutations (2013).

The vasculopathy similar to the chronic rejection of long-surviving allografts appears in the xenografts that survive for more than a few weeks (Ekser and Cooper 2010). In xenospecific CD4+ T-cell activation that occurs in chronic rejection, indirect recognition of xenoantigens is involved. Human T-cell response against pig antigens is at least as powerful as in the allotransplant response. Human CD4+ T cells mainly recognize SLA class I-derived peptides and SLA class I molecules expressed on all cell types. Identification of the immunogenic SLA class I epitopes may facilitate development of strategies inducing peptide-specific immune tolerance (Park et al. 2012a, b).

In the case of an extracorporeal perfusion of a pig liver with human blood, porcine Kupffer cells bound N-acetylneuraminic acid present on human erythrocytes and destroyed more than 90 % of them showing the other dark side of the xenotransplantation. The addition of anti-porcine sialoadhesin mAb reduced the loss of human erythrocytes over a 72-h period and prolonged pig liver metabolic function throughout the extracorporeal perfusion (Waldman et al. 2013).

Other pig-to-human xenotransplantation obstacles include molecular differences between the coagulation systems. The pig tissue factor pathway inhibitor (TFPI) is unable to neutralize the human factor Xa and cannot inhibit the direct activation of human prothrombin to thrombin. Pig thrombomodulin (TM) binds human thrombin, but this complex is ineffective in activating protein C, which promotes clotting. The von Willebrand factor interacts with higher affinity human platelet receptors, leading to elevated pro-coagulant activity. The barriers of pig-to-human transplantation are significant, but are being overcome by attempts to produce genetically engineered animals to make them indistinguishable for the human immune system.

## HAR preventing strategies

Development of methods eliminating HAR is the first step to prevent xenograft rejection. Genetically modified pig organs not expressing the Galα(1,3)Gal epitope are grafts of choice. An alternative method of reducing the number of Galα(1,3)Gal epitopes is to incorporate a gene coding for human α1,2-fucosyltransferase (HT, H-transferase) into the pig genome. Expression of HT does not allow a complete elimination of the epitopes. It is suggested to co-express α1,2-fucosyltransferase and α-galactosidase (GLA), as it removes terminal D-galactose of the epitope (Luo et al. 1999). Osman et al. reported a greater reduction in the number of the Galα(1,3)Gal epitopes on the surface of COS cells expressing both GLA and HT comparing with single transgenic cells (1997). Opposite to Osman et al., no cumulative effect of the co-expression of both enzymes was observed by our research team (Zeyland et al. 2014). Some advances in the control of complement activation were also achieved by the preparation of pig cells expressing human complement regulatory proteins (CRPs) (Zeyland et al. 2012). CD55 decay accelerating factor (DAF), CD46 membrane cofactor protein (MCP) and CD59 membrane inhibitor of reactive lysis (MIRL) are the most important complement regulators anchored in cell membranes. Murine splenocytes co-expressing human HT and CD59 showed greater resistance when exposed to the human serum in comparison to the wild type control (Costa et al. 1999). Similar observations were made in the case of CD59 expression in GTKO splenocytes of mice (Tange et al. 1997). In addition, it was demonstrated that the protective action of the co-expressed CD55 and CD59 factors was higher than that of CD55 and CD46 (Huang et al. 2001). Pigs expressing CRPs are listed in Table 1.

**Table 1:** Pig genetic modifications for xenotransplantation

Introduced/inactivated gene	Expected effect
p GGTA1	Inactivation/ prevents HAR
h CD55, CD46, CD59	Expression/ prevents HAR
h HT	Expression/ prevents HAR
h GLA	Expression/ prevents HAR
p CMAH	Inactivation/ reduces xenoantigenicity
HLA-E/h β2-microglobulin	Expression/ prevents rejection assisted by NK cells
p ULBP1	Inactivation/ prevents rejection assisted by NK cells
h CD47	Expression/ prevents rejection assisted by macrophages
h CD39	Expression/ prevents thrombosis
h TM, TFPI	Expression/ prevents delayed xenograft rejection
p CTLA4-Ig	Expression/ prevents the T-cell-mediated response
LEA29Y	Expression/ prevents activation of T-cells
h CIITA-DN	Expression/ prevents human cellular immune response
h TRAIL	Expression/ prevents cellular immune response
PERV siRNA	Expression/ prevents activation of PERV
h GnT-III	Expression/ reduces xenoantigenicity
h TNFRI-Fc	Expression/ prevents hTNF-α-mediated inflammation and apoptosis
h A20	Expression/ prevents acute vascular rejection
h HO-1	Expression/ anti-oxidative, anti-apoptotic, and anti-inflammatory effect

p – porcine, h – human, ULBP1 (UL16 binding protein 1), HLA (human leukocyte antigen), CTLA4Ig (cytoxic T-lymphocyte associated antigen4-immunoglobulin); TNFRI-Fc (tumor necrosis factor-alpha receptor I-Fc); HO-1 (heme oxygenase-1); CIITA-DN (dominant-negative mutant class II transactivator), GnT-III (N-acetylglucosaminyltransferase III), A20 (tumor necrosis factor-alpha–induced protein 3)

Some hopes for prolonging the survival time of organs are vested in the transgenic modulation of the clotting cascade by the expression of anticoagulants, or their induction or elimination of procoagulants on the xenogenic vascular endothelium. The considered options include the expression of factors such as the platelet fibrinogen receptor antagonist (GPIIbIIIa), CD62P inhibitor, CD39 (ectonucleoside triphosphate diphosphohydrolase 1), TFPI, TM, hirudin and CD73, or knocking out genes of tissue factor, protease activated receptors 3 and 4, and fibrinogen-like protein 2.

## Solid xenoorgans

It is proven that the wild type pig-to-baboon liver xenotransplantation induces platelet activation, phagocytosis and fatal hemorrhage as a consequence of thrombocytopenia. Genetically engineered pigs with the knocked out GGTA1 gene produce antibodies against Galα(1,3)Gal epitopes, which may prove an indirect confirmation of successful Gal deletion (Fang et al. 2012). Anyhow, Sharma et al. were able to detect small amounts of Gal epitopes in transgenic pigs with the homozygotic GTKO genotype (2003). In the case of GTKO, a heart transplant survived in the baboon organism for 6 months compared to 3 months in the case of a kidney graft (Cozzi et al. 2000; Kuwaki et al. 2005; Tseng et al. 2005; Yamada et al. 2005). Kim et al. transplanted livers from GTKO pigs to baboons under immunosuppressive treatment, suggesting that thrombocytopenia after xenotransplantation may be overcome by aminocaproic acid (Amicar®) therapy. One baboon, showing no histopathological evidence of rejection, died after 6 days from uncontrolled bleeding. Aminocaproic acid treatment helped two other animals to survive 2 and 3 days longer after xenotransplantation (2012). To prevent thrombosis and inflammation that occur after solid organ xenotransplantation, transgenic pigs overexpressing anticoagulant, antiinflammatory and/or cytoprotective factors may be produced. Transgenic mice expressing human endothelial protein C receptor (hEPCR) were protected against warm renal ischemia reperfusion injury. Hearts from these mice survived longer after heterotropic transplantation in C6-deficient rats showing less edema and hemorrhage (Lee et al. 2012). Platelet aggregation plays a key role in the development of thrombotic microangiopathy in primate recipients of pig xenografts. Collagen, adenosine diphosphate and thrombin induce greater platelet aggregation in humans than in baboons, whereas adenosine diphosphate and thrombin induce more platelet aggregation in cynomolgus monkeys than in baboons. Over all, thrombin among its agonists investigated by Iwase et al. was responsible for the highest level of platelet aggregation in all the above mentioned species (2012). Thus thrombin formation plays a key role in the activation of coagulation and it is suggested that organs from GTKO pigs with human TM may lead to a decreased immunological response.

Xenotransplantation of transgenic organs from pigs expressing human complement regulatory factors did not activate the complement cascade, showing effectiveness of the strategy in overcoming HAR. Hearts from transgenic pigs expressing DAF and CD59, transplanted into baboons, showed less vascular injury and functioned for prolonged periods when compared to hearts from wild-type pigs (McCurry et al. 1995). CD59/DAF pig hearts transplanted into baboons immunosuppressed with cyclosporine, methylprednisone or leflunomide/mofetil mycophenolate functioned for 85–130 h. Wild-type pig hearts survived for only 20–80 min. Hearts from transgenic animals showed less HAR, but were characterized by acute vascular rejection with a high level of IgG deposits (Chen et al. 1999). Cynomolgus monkeys and baboons under immunosuppressive treatment received kidneys or hearts from pigs expressing human DAF. The presence of hDAF fully prevented kidney xenograft in cynomolgus monkey from HAR and partially grafted hearts in cynomolgus monkeys or baboons (Schuurman et al. 2002). McGregor et al. performed heterotopic heart transplantations from hCD46 transgenic pigs to baboons using a combination of therapeutic agents. Two out of seven grafts were lost as a result of rejection. The median graft survival time was 96 days. No consumptive coagulopathy, cellular infiltration or posttransplantation lymphoproliferative disorders occurred (2005). Mohiuddin et al. suggested a critical role for B cells in the mechanisms of elicited non-Galα(1,3)Gal antibody and delayed xenograft rejection. They reported a significant extension of heterotopic hCD46/GTKO pig cardiac xenograft survival in specific pathogen free baboons. Peritransplant B-cell depletion in the context of an established immunosuppressive regimen prolonged GTKO/hCD46 graft survival up to 236 days (maximum). B-cell depletion persisted for over 2 months (2012).

Hyperacute rejection, acute humoral xenograft rejection and acute cellular rejection in case of solid genetically modified xenoorgans can be partially controlled by an administration of immunosuppressive therapies, but the development of graft vasculopathy in the case of chronic rejection is still a big challenge.

## Islet xenotransplantation

Non-vascularized tissue transplantation is believed not to provoke classical HAR rejection. Porcine islet endocrine cells express low levels of the Galα(1,3)Gal epitope, which makes them resistant to most xenoreactive antibodies (Dor et al. 2004). The islet isolation procedure removes most of the vasculature and moreover, after transplantation islet revascularization is of the recipient origin. In vitro exposure of porcine Langerhans islets to human whole blood results in a rapid inflammatory reaction followed by macroscopic coagulation, complement activation, consumption of platelets and infiltration of immune cells (mainly T cells) (Bennet et al. 2000). Instant blood-mediated inflammatory reaction (IBMIR) is characterized by kinetics similar to HAR, but antibody deposition is not observed. Porcine islets that survive IBMIR undergo cell-mediated rejection. A broad spectrum of factors, i.a. the implementation site or immunosuppression treatment, can affect porcine islet survival after transplantation to a primate recipient. The portal vein is the site of choice also in allotransplantation, but unfortunately it is also risky because of the islet graft direct contact with blood leading to IBMIR and early cell loss. Islet damage involves membrane leakage, antibody deposition, complement activation, formation of the C5b-9 membrane attack complex and mitochondrial dysfunction. The gastric submucosal space, subcutaneous fat, intramuscular (e.g. parathyroid glands), intraperitoneal and omental sites are investigated as alternatives for the portal vein. In spite of the massive loss, estimated at up to 80 %, the remaining cells are able to control blood sugar and sustain normoglycaemia. An immunosuppressive compilation of anti-IL2R and anti-CD40L monoclonal antibodies, fingolimod or tacrolimus, everolimus and leflunomide, supported the survival of wild-type porcine islets in pancreatectomized cynomolgus macaques for longer than 180 days. This resulted from suppressing indirect activation of T cells, induction of IgG antibodies, expression of proinflammatory cytokines and invasion of infiltrating mononuclear cells (Hering et al. 2006). Also temporal T-cell depletion, as in the clinically practiced anti-thymocyte-globulin therapy, combined with human alpha-1-antitrypsin administration, may promote islet xenograft acceptance (Ashkenazi et al. 2013). Luan and Iwata encapsulated rat islets with agarose-immobilized microbeads of the inhibitor of the classical and alternative complement activation pathways, called soluble complement receptor 1 (sCR1). After transplantation to mice not treated with immunosuppressants, grafts survived 22 days longer than the control islets showing some protective effect of sCR1 (2012). Although the xenogeneic islet microencapsulation prevents direct contact with the host immune system, the observed graft infiltration by host macrophages leads to pericapsular fibrotic overgrowth in the case of encapsulated fetal porcine islet-like cell clusters transplanted into the peritoneal cavity of immunocompetent mice (Vaithilingam et al. 2013). Macrophages were also responsible for a function collapse of the encapsulated human islets in Ca2+/Ba2+ alginate microbeads transplanted to diabetic Balb/c mice (Qi et al. 2012).

As an alternative to the immunosuppressive strategies and islet encapsulation, some transgenic attempts to reduce T-cell mediated immune response were undertaken. Transgenic pigs expressing the human tumor necrosis factor-alpha-related apoptosis-inducing ligand (TRAIL) have been produced (Klose et al. 2005). The main biological role of TRAIL is to kill tumor and virus-infected cells. DR4 and DR5 — death receptors highly expressed on activated lymphocytes, induce apoptosis after engagement by TRAIL. Adenocarcinoma cells and mastocytoma tumors expressing TRAIL avoided attack of tumor-specific T cells and tumor-infiltrating macrophages, respectively. Expression of human TRAIL on porcine dendritic cells in vitro attenuated the human primary T cell response (Kemter et al. 2012).

## Other tissue/cellular xenotransplantations

Parkinson’s disease (PD) is a brain disorder related to the loss of dopamine producing cells. A possible therapeutic solution would be to implement PD patients with pig mesencephalic dopaminergic-enriched cells. This strategy was applied in *Macaca fascicularis* after PD induction. Twelve PD monkeys received human embryonic neural precursor cells expressing CTLA-4-Ig (cytoxic T-lymphocyte associated antigen4-immunoglobulin), which can bind to B7 molecules expressed on dendritic cells and activate the tryptophan catabolic pathway that can lead to indirect inhibition of lymphocyte activation and T cell death. Control studies showed a highly significant recovery of spontaneous locomotion in all grafted animals, which can be partially explained by a fragmentary restoration of dopaminergic activity detected by PET scans in at least six animals. This progress in locomotor activity was observed even after more than 15 months. Histological analyses showed the existence of porcine grafts composed of dopaminergic, serotoninergic and GABAergic differentiated neurons and various glial components, which is very promising for humans suffering from PD (Badin et al. 2010).

Allotransplantation of the liver as a whole organ is limited by the shortage of human donors. Xenotransplantation of pig hepatocytes could provide patients with hope of alleviating metabolic deficiencies and supporting the damaged organ function. Nagata et al. transplanted 1–2 billion hepatocytes into spleens of cynomolgus monkeys under immunosuppressive treatment. Xenogeneic hepatocytes functioned for more than 80 days after infusion and for more than 253 days after retransplantation with no perceivable influence on the grafts’ survival (2007).

Corneal blindness is still a highly prevalent disease. In the developed world corneal allotransplantation is widely available, but globally the demand for corneas far exceeds their supply. The cornea as not immediately vascularized, immunologically privileged tissue is believed to be more promising than solid xenoorgans after transplantation. Immune privilege is an evolutionary adaptation that protects structures (i.e. the brain, ovaries, testes, adrenal cortex) from damage caused by an inflammatory immune response. The cornea, anterior chamber, vitreous cavity and sub-retinal space are immune-privileged in the eye. The cornea is avascular and the aqueous humor contains several factors with anti-complement activity. Wild type Wuzhishan pig corneal grafts were rejected within 15 days without any signs of HAR after orthotopic penetrating xenotransplantation into the eyes of non-immunosupressed rhesus monkeys. Rejection was delayed for more than 4 months by conjunctival injection with betamethasone. The use of lamellar corneal xenografts maintained corneal transparency for more than 3 months without steroid treatment to the eye (Pan et al. 2007). Kelley et al. reported that five of six small (6.5 mm) human corneal grafts in rhesus monkeys survived over 6–9 months. Five out of six larger grafts (9.5 mm) had been rejected by the end of the 6-month period. These findings suggest that graft size influences survival (Kelley et al. 1984). Jie et al. checked the survival of corneal graft from Wuzhishan pigs in rhesus monkeys after xenotransplantation following donor bone marrow transplantation (BMT). Animals were tested for chimerism, mixed lymphocyte reaction (MLR) and immunoglobulin and the complement levels in the serum. The mean survival time was 10 days longer than in the control monkeys that also received intravenous injection of cyclophosphamide (CP), but did not undergo BMT. Immunoglobulin and complement levels in the serum showed a downward trend. Histopathological examinations demonstrated that the corneal xenografts had minimal inflammatory cell infiltration and no eosinophil infiltration in monkeys after BMT (2013).

Adipose-derived mesenchymal stem cells (AD-MSCs) are thought to be promising sources in regenerative medicine of eye diseases, especially to treat retinal blindness. Human AD-MSC were xenotransplanted into the eyes of rats and assessed for survival during a 6-month period. AD-MSCs were detected in the vitreous humor for up to 90 days, but they were also integrated into the ocular tissues. Some of the cells crossed the blood-retina barrier and were detected in the spleen, indicating the problem of stem cell migration to non-target sites (Haddad-Mashadrizeh et al. 2013a, b). The same problem was observed in the case of human AD-MSCs crossing the blood barrier after xenotransplantation to the rat brain (Haddad-Mashadrizeh et al. 2013a, b). This should be limited by the use of immune privilege cells and tissues for implantation.

Pig RBC (red blood cell) diameters and counts are similar, although the average life span is shorter than that of human RBCs. Porcine hemoglobin with a similar three-dimensional structure shares 85 % sequence identity with its human counterpart. Pig RBCs do not express swine leukocyte antigens, thus reducing immunogenicity. Removing Galα(1,3)Gal epitopes by GLA treatment prevented binding of baboon and human antibodies to pRBCs in vitro. GLA treated pRBCs were undetectable for baboon immunological system for at least several hours. Previous treatment of wild type pRBCs with GLA and neuraminidase would be required to eliminate the risk of a hemolytic reaction after transfusion (Cooper 2012). In vitro, native human or baboon serum IgM binding was detected on GTKO pRBCs, but it was significantly lower than binding to wild type pRBCs, while IgG binding was absent or minimal. In vivo studies showed that GTKO pRBCs are rapidly phagocytosed from the primate circulation by a mechanism not involving anti-Gal antibodies (Rouhani et al. 2004). GTKO pRBCs are significantly more compatible than ABO-incompatible human RBCs and wild type pRBCs, but they are not comparable with ABO-compatible human combinations (Long et al. 2009). In the context of blood transfusion, production of GTKO pigs with added hCD47 expression is being considered. The limited compatibility (73 %) of amino acid sequences between the pig and human CD47 may contribute to phagocytosis of xenogeneic cells by macrophages that can even act in the absence of antibodies or complement opsonization. SIRPα (the signal regulatory protein α) is a receptor on macrophages that interacts with CD47 and prevents autologous phagocytosis. Expression of human CD47 on porcine cells provides inhibitory signaling to SIRPα on human macrophages (Ide et al. 2007).

## Final conclusions

When we consider xenotransplantation in the context of its safety as a clinical approach, we think of minimizing the risk of infectious agent transmission through this procedure. The considerable phylogenetic distance between humans and pigs may limit the prevalence of infections caused by zoonotic pathogens (bacterial, viral and parasitic), especially when animals are bred under strictly controlled conditions in selected, pathogen-free herds. Specific knockouts and short interfering RNAs specific for viral sequences have been proposed as a strategy of virus eradication from the pig herds (Fiebig et al. 2003; Miyagawa et al. 2005). During the past decade, no transmission of infectious agents to humans exposed to live porcine cells and tissues was recorded. Nevertheless, the greatest hazard is still seen in porcine endogenous retroviruses (PERVs), classified on the basis of the differences in receptor recognition to the A, B and C classes. PERV-A and PERV-B can infect human cells in vitro, but PERV-C lacks this capacity. Recombination between PERV-A and PERV-C is possible. Although this recombinant virus can infect human cells, no PERV-A/C virus infection was reported in humans. Pigs with a low expression of PERV-A and PERV-B and free from PERV-C should be used for xenotransplantation. Another virus — porcine cytomegalovirus (PCMV) — was detected in primates after xenotransplantation, but it did not lead to invasive disease of the host. When it comes to the porcine lymphotropic viruses (PLHVs), only PLHV-1 is associated with a lymphoproliferative syndrome, but such a disorder has not been observed after pig-to-primate xenotransplantations (Mueller et al. 2004).

There are four weaknesses associated with strategies involved in preventing xenograft rejection: the use of immunosuppression and inducing the tolerance, encouraging non-infectious or latent pathogens to become malicious. GTKO pig cells release viruses without Galα(1,3)Gal epitopes, which makes them less visible for immunological system. Expression of hCRPs on the surface of transgenic pig cells may contribute to a weakening of the complement defense against infections and at the same time encourage pathogens to adopt human complement regulatory proteins as the infectious binding receptors (Weiss 1998).

In conclusion, only pigs with numerous genetic modifications obtained through interbreeding of organisms with single modifications or through cotransfection with several gene constructs will become a real reservoir of organs for medical purposes. Crossbreeding of genetically modified pigs available for research (Table 1) give us hope for xenotransplantation to become a medical procedure commonly used.
